# Professional Relations between Dentists and Doctors

**Published:** 1909-01-09

**Authors:** 


### Dental Department.

### PROFESSIONAL RELATIONS BETWEEN DENTISTS AND DOCTORS.

There has been some controversy in the medical and dental journals recently on a point of considerable interest to medical practitioners and dentists. A certain general practitioner recommended a patient to a dental surgeon for treatment. The dentist examined the patient, and suggested treatment which involved the extraction of several teeth, and an anæsthetic being required he decided on nitrous oxide gas, which he wished given by the continuous nasal method. The patient expressed a wish that his own medical attendant—who recommended him to the dentist—should give the anæsthetic. "Oh! no," said the dentist, "I always employ a specialist to give anæsthetics for me." On this coming to the ears of the medical man he was very indignant, and wrote to the papers accusing the dentist in no measured terms of want of ordinary politeness.

In trying to take an unbiassed view of the case, we must remember that there are a great many general practitioners who have little if any experience in giving $N_2O$. Rightly or wrongly, the dentist has to bear the brunt of any want of success either with the operation or the anæsthesia; the operation takes place at his house, his share of the responsibility is the greater, and it is his practice that will suffer should any untoward result take place. This being so, it is small wonder that he judges it important in the interest of both his patient and himself; to exercise some care in the choice of his anæsthetist.

The question of anæsthetics is very much to the fore just now, and while we are in the throes of some process of evolution, we have to assume that all medical men are practical and competent anæsthetists, unless we have experience to the contrary. We think the dentist in the above instance was to blame, and was certainly wanting in tact; the patient being under the care of the medical man, who had more right to give the anæsthetic? It was not the dentist's place to assume that the doctor was incompetent to give any anæsthetic in any way desirable—as a matter of fact the doctor was well skilled in this particular form of anæsthesia.

What the dentist should have done under the circumstances was to write to the doctor thanking him for the recommendation and informing him briefly of the condition of the patient's mouth; to suggest certain treatment, that an anæsthetic would be required, and that he would prefer nitrous oxide gas by the continuous nasal method. He might then have inquired whether the doctor would like to give the anæsthetic himself, or whether another anæsthetist should give it, and if so would the doctor care to be present at the operation. In this way if the doctor did not care to give the anæsthetic he could retire gracefully from the position, the patient's wishes could be met by the doctor being present at the operation, and the dentist would to some extent safeguard himself; for it is unlikely that the doctor would have insisted on giving the anæsethetic after such a letter, had he not felt competent to do what was required, as we mentioned the doctor in question was.

More and more do doctors and dentists come into close professional relationship, and the exercise of tact and courtesy makes for smooth working, and, for both are busy men, it saves time and misunderstanding. Many small points are constantly cropping up between doctor and dentist, and by the exercise of tact and good feeling, much worry and irritation may be avoided. Thus, a lady brought her somewhat delicate-looking little girl, aged 12, to a dentist, seeking advice about a quite disfiguring irregularity of her teeth. The dentist strongly advised immediate regulation of the teeth, which involved the extraction of four badly-decayed first permanent molars. The child had apparently no organic disease at the time the operation was advised. The dentist left the lady to make arrangements with her medical adviser for the administration of the anæsthetic. All the dentist heard of the case again was a short note from the mother saying that her doctor did not advise anything being done at present.

This was disconcerting to the dentist, and showed a want of courtesy and knowledge of the fitness of things on the part of the doctor. He was not in a position to judge of the urgency or necessity of the treatment suggested; he might have written a short note to the dentist before giving an opinion to the mother, stating his reasons for wishing to postpone the operation, and asking the dentist's opinion.

Again, a dentist did a considerable amount of conservative dentistry for a lady, who almost immediately afterwards developed a large painful swelling near the angle of the jaw, with considerable general disturbance. The first the dentist heard of the case was on meeting the lady's husband and inquiring after her health. Being told that she was ill in bed, in the hands of the doctor, in great pain, and with a large swelling at the angle of the jaw, and unable to open her mouth, the dentist naturally had considerable misgiving as to the result of his treatment. He suggested to the husband that if the doctor were agreeable he would call and see the lady, and ascertain, if possible, whether the trouble arose from the teeth; the medical man having confessed himself unable at the time to determine the cause of the swelling. Now a short note from the doctor stating the conditions, and suggesting that the dentist should call and examine the patient, with a view to discovering whether the teeth were at fault, would have saved a considerable amount of irritation, and, to say the least of it, would have been an act of courtesy.

If a doctor sends a patient to a dentist for treatment, as a matter of courtesy the dentist should write to the doctor, briefly indicating the conditions found, and the treatment suggested. Should a general anæsthetic be required, it is the rankest discourtesy on the part of the dentist to call in another medical man to give the anæsthetic without first ascertaining whether the doctor who recommended the patient wishes to give the anæsthetic himself.

Again, if a dentist is consulted by a patient, and a line of treatment agreed upon, and for some reason or other that patient sees a medical man, it is the rankest discourtesy on the part of the medical man to induce that patient to leave the dentist he had consulted, and to persuade him to go to some other dentist, no matter how friendly the doctor may be with the latter. Such cases have happened again and again, and have led to much ill-feeling.

Both doctors and dentists should avoid to the utmost allowing the layman to inveigle them into giving an adverse opinion of the ability and professional conduct of a brother-practitioner; for there is nothing the lay mind enjoys so much as pitting the opinion of one medical man against another, and inducing one to criticise another adversely.

As a final point between doctors and dentists, we may say a word about fees. When a medical man gives an anæsthetic for a dentist, if the patient is also a patient of the medical man, the latter usually sends his account direct to the patient; if, on the other hand, the dentist calls him in to anæsthetise a stranger, then the dentist pays him.

If a medical man consults a dentist about work for his own mouth or for members of his family, and *vice versa* if a dentist requires the services of a medical man for himself, or the members of his family, a difficult question of payment for services arises. While medical practitioners do, no doubt, give one another their services freely, and dental practitioners are willing to do work for each other without charge, and while dentists would wish to be able to give their services freely to their brother medical practitioners and their families, this may entail great hardship on the dentist, for while the dentist could only avail himself of the services of one medical man for himself and his family, he might find himself attending to several medical men and their wives and families. Moreover, dental attendance almost always entails out-of-pocket expenses in material, which may, indeed, be very heavy, for if the attendance is to be complete it will almost certainly involve the use of a considerable amount of costly appliances.

We think that a dentist might perhaps attend to those medical men and their families with whom he is in close professional relationship; such attendance should be freely given as far as simple extractions and fillings go, and when precious metal and costly material are used a fee should be charged to cover the cost of the material used; and other medical men might be charged a reduced fee.

